# Does the use of face masks during the COVID-19 pandemic impact on oral hygiene habits, oral conditions, reasons to seek dental care and esthetic concerns?

**DOI:** 10.4317/jced.57798

**Published:** 2021-04-01

**Authors:** Célia-Regina-Maio Pinzan-Vercelino, Karina-Maria-Salvatore Freitas, Valquiria-Mendes-Pereira Girão, Daniella-de Oliveira da Silva, Renan-Morais Peloso, Arnaldo Pinzan

**Affiliations:** 1 DDS, MSc, PhD. Professor, Department of Orthodontics; University Ceuma (CEUMA), São Luís, MA, Brazil; 2 DDS, MSc, PhD. Professor, Department of Orthodontics; Uningá University Center (UNINGÁ), Maringá, PR, Brazil; 3 DDS. MSc Student; University Ceuma (CEUMA), São Luís, MA, Brazil; 4 DDS, MSc. PhD Student; University Ceuma (CEUMA), São Luís, MA, Brazil; 5 DDS. MSc Student; Uningá University Center (UNINGÁ), Maringá, PR, Brazil; 6 DDS, MSc, PhD. Professor, Department of Orthodontics; Bauru Dental School, University of São Paulo (FOB – USP), Bauru, SP, Brazil

## Abstract

**Background:**

To evaluate the impact of the use of face masks on oral hygiene habits; oral conditions self-perception; reasons to seek dental treatment; and esthetic concerns.

**Material and Methods:**

1346 participants answered a web-based survey with questions related to the aims of the study. Descriptive statistic was performed and the responses were analyzed with chi-square test and regression analysis.

**Results:**

With the use of masks, toothbrushing frequency decreased significantly, and people are significantly less concerned about oral hygiene. The number of subjects that reported to have halitosis increased significantly and this was associated with a decrease in toothbrushing frequency. The greatest complaints of subjects were teeth color and alignment. The prevalence of bruxism increased significantly. Overall, 94.1% considered that esthetics is important to seek dental care even with the use of masks, and 84.2% are maintaining usual periodic dental care. People are significantly less concerned with their smile and dental esthetics with the use of masks.

**Conclusions:**

With the use of face mask, people must be motivated regarding maintaining oral hygiene habits. Some respondents will seek dental care only when masks were no longer necessary, therefore dentists must be aware of a reduction in the volume of patients.

** Key words:**Dental health surveys, esthetics, oral hygiene, dental care, COVID-19, behavior.

## Introduction

The novel coronavirus (SARS-CoV-2) that causes the COVID-19 initiated in December of 2019 in China and triggered a global outbreak, with still no currently effective pharmacological intervention or vaccine available. Countries are maximizing their efforts to combat this pandemic and to minimize infection (1). Since interpersonal transmission occurs mainly via respiratory droplets and contact transmission, the governments worldwide have adopted many precautionary measures and the use of face masks in public places has been compulsory or recommended. From June 5, 2020, the World Health Organization (WHO) advised that to effectively prevent COVID-19 infection in areas of community transmission, governments should encourage the general public to use masks in specific situations and settings as part of a comprehensive approach to suppress SARS-CoV-2 transmission (2).

Face masks are physical barriers. They are simple, cheap and efficient in reducing short distance transmission through direct or indirect contact and droplet emission (1-3). The masks may be worn at home (particularly by the person showing symptoms) and also in public places (3). The purpose of wearing masks is to prevent infected people from transmitting the virus to others and offering protection to healthy people against infection (2). Despite the indication of use, its impact on social life, behavior and oral hygiene habits is still unknown.

At the least decades, people have shown a desire for a more pleasant physical appearance. Many patients seek dental treatment with esthetics complaints (4,5). The mouth is one of the first features observed during interpersonal interactions (6,7). However, the masks cover the lower third of the face so people may have been neglecting aspects related to oral hygiene and dental esthetics.

The COVID-19 pandemic and social distancing are causing economic effects on dental practices (8) and influencing people’s dental care-seeking behavior (9,10). Infection risk in the dental environment is a serious problem for both professional practitioners and patients (11). Reductions in activities were observed at prevention, periodontics, prosthetics (8) and maxillofacial surgery practice.(12) The knowledge of the changes in patients’ esthetics complaints, perceptions and habits with the use of masks is important for the clinicians’ to be aware of what to expect with the dental offices reopening.

Therefore, the purpose of this study was to evaluate the impact of the use of face masks during COVID-19 pandemic on oral hygiene habits; dental, smile and oral conditions self-perception; current reasons to seek dental care; and the importance attributed to teeth and smile esthetics.

## Material and Methods

This cross-sectional study received approval from the local human research ethics committee (protocol number: 4.079.866). This survey was carried out in Brazilian adult individuals (more than 18 years of age) who were wearing face masks in the last 30 days.

An electronic survey (Google Forms) was used to generate a web-based platform for the responses. The online survey was available for responses from June 10 to June 20, 2020. In the introduction of the questionnaire, the informed consent approved by human research ethics committee was described, and the subjects were informed about the objectives. The questionnaire was anonymous; no personal identification was required. A link to the survey was posted on Facebook, Instagram, Twitter and sent by WhatsApp by the authors. Since this was a web-based open survey, a sample size calculation was not performed and it was not possible to determine a response rate.

The questionnaire contained 41 multiple-choice questions oriented in the following directions: oral hygiene habits; dental, smile and oral conditions self-perception; reasons to seek dental treatment; and the importance of esthetics as a motivating factor for dental treatments (Appendix).

Data regarding participants’ characteristics (sex, age, region of residence, level of education, dental background and use or not of orthodontic appliances) were collected.

The first set of questions about oral hygiene habits comprised: frequency of toothbrushing, flossing and use of mouthwash or dental rinse per day, before and with the use of masks, and concern with oral hygiene.

To evaluate dental, smile and oral conditions self-perception, they were asked about halitosis; smile; dental complaints; gingival bleeding; oral health and bruxism (clenching or grinding teeth during sleep and/or wakefulness).

The third set of variables was about the reasons to seek dental care: the importance of dental and smile esthetics as reason for dental treatment, dental care seek in the last 30 days and its purpose; the need for emergency dental care during quarantine; concern about the frequency for seeking dental care with the use of masks.

In order to evaluate the importance of teeth and smile esthetics, the participants were asked about: concern about teeth and smile esthetics before and with the use of mask; if they felt better about their teeth and smile wearing masks; if they missed looking at other people’s smiles; and the importance of the smile.

A structured questionnaire was developed and tested on a pilot population before its administration in this study. The pilot study was undertaken with 30 laypeople to evaluate the clarity of the questions and the language used. Some words were rewritten with synonyms so that the individuals were more likely to understand. The pilot study participants were not included in the main study.

-Statistical analysis

To evaluate intrarater agreement, question 30 was duplicated in the questionnaire. This question was chosen for repetition because it has only two responses (yes or no), and was a critical and important question in the survey. The answers to this duplicate question were analyzed using Kappa statistics. The result showed a coefficient of 0.93, indicating an excellent agreement.

Descriptive statistics were performed with percentages. Chi-square tests were used to compare answers before and with the use of mask, males and females, laypeople and subjects with a dental background. Data analysis also included binomial and multivariate logistic regressions considering the concerns with oral hygiene and dental and smile esthetics as the dependent variables and several independent variables such as sex, age, educational level and oral hygiene habits.

Statistical analysis was performed with Statistica software (version 10.0, Statsoft, Tulsa, USA) and results were considered significant for *P*<0.05.

## Results

A total of 1358 individuals accessed the link, and 1346 agreed to participate and answered the questions. The demographic variables are shown in Table 1.

**Table 1:**
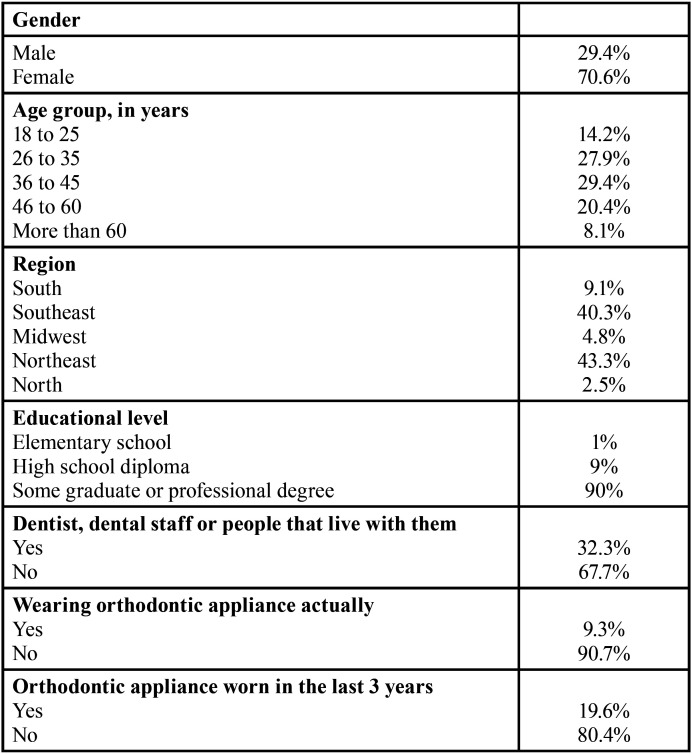
Demographic variables.

-Oral hygiene practice

Data showed that, with the use of masks, significantly more subjects were brushing their teeth fewer times per day, and people were significantly less concerned about oral hygiene (Table 2).

**Table 2:**
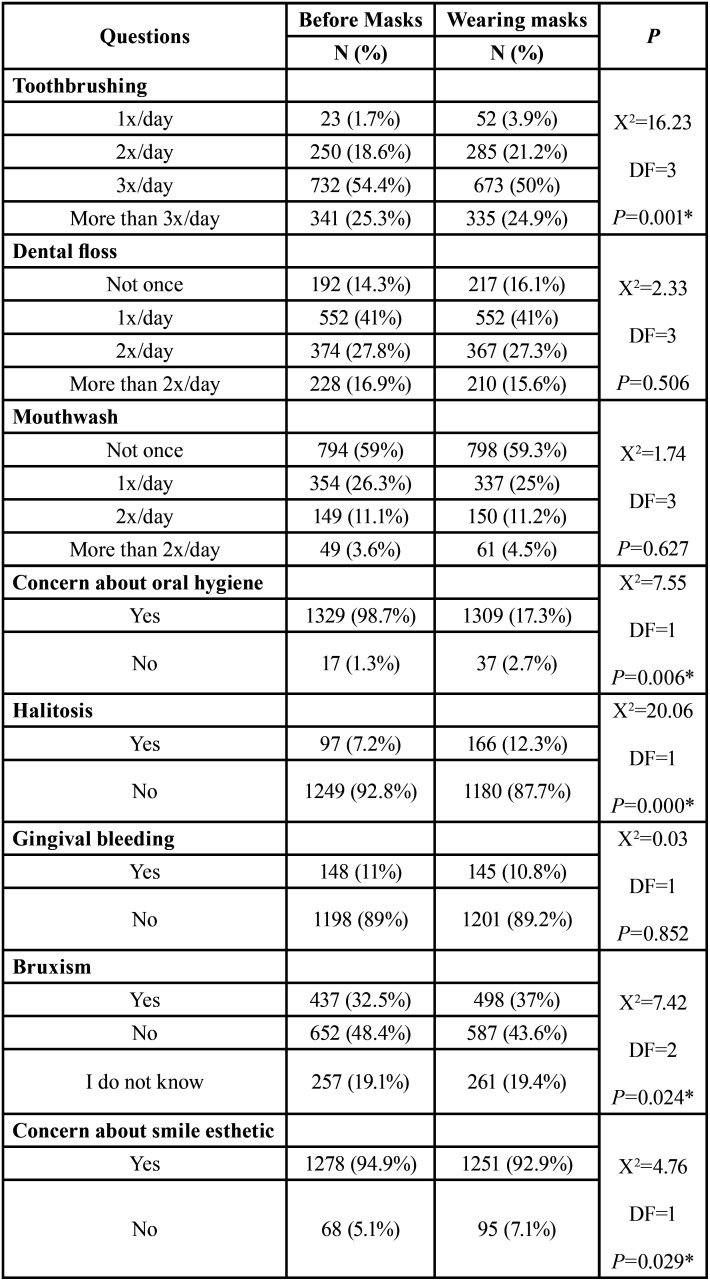
Comparison of responses to oral health questions before and with the use of masks (chi-square tests).

Respondents with a dental background (dentists, dental staff or living with some of them) were significantly more concerned with oral hygiene, and their oral hygiene habits were significantly better both before and with the use of masks than the other individuals that answered the questionnaire (*p*<0.001).

There was no difference in oral hygiene practice between males and females. The younger subjects and the respondents with only elementary school showed lesser care with oral hygiene (*p*<0.001).

Dental, smile and oral conditions self-perception

The number of subjects that reported to have halitosis increased significantly (Table 2). There was a significant association between the decrease of toothbrushing frequency and the presence of halitosis (*p*<0.001).

Most of the subjects reported to have a pleasant smile (51.3%), 25.4% an accepTable smile, and 19.7% considered their smile very pleasant. Only 2.9% reported having an unpleasant smile and 0.7%, a very unpleasant smile.

The greatest complaints of subjects were teeth color (56%) and teeth alignment (30.2%). Regarding the teeth color, 46.5% considered accepTable, 42.2% reported to be pleasant, 6.5% considered it very pleasant and only 4.8% considered it unpleasant. From these people that considered their teeth color unpleasant, 93.8% think it is too dark and 4.5% think their teeth are yellowish. About teeth positioning, 45.5% considered pleasant, 32.7% accepTable, 14.6% very pleasant and 7.3% unpleasant. Between the subjects that answered that their teeth were unpleasant, 61.2% reported that this was due to crowding and 38.8% due to diastema.

Gingival bleeding was similar before and with the use of masks (*p*=0.852) (Table 2). Regarding oral health, 44.9% and 37.1% considered having good or very good oral health, respectively; 9.4% considered excellent and 8.6% considered it deficient or very deficient.

The prevalence of bruxism (clenching or grinding teeth during sleep and/or wakefulness) increased significantly (*p*=0.024), and 47.8% of the subjects that related clenching or grinding teeth were feeling myofascial pain.

Reasons to seek dental treatment

The great majority (94.1%) considered that smile and dental esthetics are important to seek dental care; 84.2% answered that, with the use of masks, should consult the dentist with similar assiduity than before; 10.5% answered that more visits to the dentist are needed and 5.3%, lesser visits than before. Overall, 89.5% answered that they would seek dental care due to smile and dental esthetics complaints even with the use of masks.

Only 10% of the respondents needed to seek emergency dental care during quarantine, for several different reasons, and the most mentioned was tooth pain (24.6%).

Dental care was scheduled by 18.1% of the respondents in the last 30 days (the authorities already had permitted dental care attendance when the subjects were inquired). The reasons for these appointments were: continuing a dental treatment started before the pandemic (26.6%), replace fillings (25.8%), regular dental appointment (24.2%) and orthodontic treatment appointment (22.5%).

Almost half of the subjects (47%) had already undergone teeth bleaching, and 73.1% would like to whiten their teeth even during the period of masks use.

Between subjects that answered that their teeth are unpleasant, 17.3% would use orthodontic appliances only when masks were no longer necessary.

 Before the face masks use, 29.9% would like to replace fillings (61.8% on posterior teeth, 11.2% on anterior teeth and 27% on both). Between them, 76.9% answered that they would go to the dentist to replace fillings even with the use of masks.

Only 6.5% of the subjects sought a dentist because of teeth clenching/grinding during sleep and/or wakefulness after the beginning of the COVID-19 pandemic.

The importance attributed to teeth and smile esthetics

With the use of masks, the number of subjects with no concern about smile esthetics increased significantly (21.5% before, 28.2% with masks, *p*<0.001) (Table 2). There was no significant difference in the concern with smile esthetics between participants with a dental background and the other subjects both before (*p*>0.05) and with the use of masks (*p*>0.05). There were no differences between the regions (*p*>0.05). Women, younger people and subjects that had completed high school or had university or professional degrees reported more concern with smile esthetics (*p*<0.001).

Even with the use of masks, 81.2% did not feel better about teeth and smile esthetics and 82.8% reported to miss looking at people’s smiles. Women miss significantly more looking at other people’s smiles (*p*<0.001). The great majority of the respondents (92.2%) think that it is important to look at the smile of people.

Discussion 

Many daily habits were altered due to the COVID-19 pandemic and one of them is the use of masks. This study raises important information regarding the present situation, helping the dentists to provide orientations to avoid an increase in oral diseases and in preparing appropriate countermeasures during the pandemic.

When the questionnaire was sent to the subjects, the use of face mask had been advised or compulsory in many cities and states in Brazil for more than 40 days, and the coronavirus pandemic was at the outbreak’s rising curve.

Data showed that the subjects were brushing their teeth fewer times per day with the use of masks, and people are less concerned about oral hygiene. The results obtained are of great concern because toothbrushing is considered a reliable means of plaque control (13). The decrease of toothbrushing frequency is associated with a higher risk for the incidence or increment of new carious lesions (14). Moreover, this practice is important to maintain periodontal health (13). Oral diseases such as dental caries, gingivitis and periodontal infection may affect oral health and can lead to teeth loss. Government and Dental Council actions must be encouraged to motivate oral hygiene habits during the period of use of masks, mainly to younger adults and people with only elementary school completed.

The number of subjects that reported to have halitosis increased significantly with the use of masks, and there was a significant association between toothbrushing less time per day and the presence of halitosis, corroborating the findings of Al-Ansari *et al.* (15) This data emphasizes the need for actions to encourage people to maintain good hygiene oral habits. Regular and effective toothbrushing and flossing can significantly reduce halitosis, especially in people with poor oral hygiene (16).

Even with the use of masks, teeth color followed by teeth alignment were the main complaints of the subjects. This result corroborated previous studies that showed that dental bleaching and alignment were most of the complaints of patients who visit dental offices (4,5).

The prevalence of self-perception bruxism increased significantly after the beginning of the COVID-19 pandemic. This result probably is related with the increase in anxiety and stress (10,17), since these problems are associated with bruxism (18,19). Dentists should be aware during anamnesis and clinical examination regarding the signs of this parafunctional activity to avoid worsening of its condition.

Most participants answered that they would seek dental care due to smile and dental esthetics, even with the use of masks, and also considered that they should consult the dentist with similar assiduity than before the use of masks. Recent studies demonstrated a decrease in psychopathological symptoms after the obligation to wear masks in public spaces (20) and workplaces (21). Face masks increased the level of perceived self-protection as well as improved mental health well-being (20). Besides, other measures as alcohol gel available at reception, medical caps and to avoid crossing other patients at reception were considered by patients as important precautionary measures to avoid contamination by the coronavirus in dental offices (10,22). Patients are conscious that these recommendations are important, and to follow them will improve the patient/dentist confidence and relationship.

However, only 18.1% scheduled dental care in the last 30 days, and the most frequent answers for dental care appointment were to continue a dental treatment started before the pandemic, to replace fillings, regular dental care and orthodontic treatment. Peloso *et al.* (10) observed that patients undergoing dental therapy showed greater concerns about their treatments and probably would not miss an appointment to avoid impairing the treatment outcome. Patients under treatment were also more concerned about an increase in the treatment duration than those under no treatment (10). It is important to consider that a negative perception of dental/smile esthetics is not necessarily followed by seeking correction of esthetic problems (4).

Some participants will seek dental care only when masks were no longer necessary. Probably this finding may be related to feelings of anxiety and fear (10). People are reluctant to go out and are staying home, less willing to go to dental offices due to the fear of contracting COVID-19 (9,10,23). So, dentists must be aware of a reduction in the volume of patients and a possible financial impact, needing to consider practice re-organization to maintain profitability (8).

Only 10% of the respondents needed to seek emergency dental care during the quarantine. Previous studies demonstrated that COVID-19 significantly influenced people’ dental care-seeking behavior, showing that fewer patients visited the dental urgency after the beginning of the pandemic (9,23,24). The most mentioned reason to seek emergency dental treatment in the present study was tooth pain, corroborating with the finds observed by Bai *et al.* (24).

The results demonstrated that people were significantly less concerned with their smiles with the use of masks. It is probably because masks cover the lower third of the face. Furthermore, the increase of feelings of anxiety and fear due to the COVID-19 pandemic may have affected their complaints since people may be concerned about other problems relating to health, financial/economic aspects, and social distancing (10).

Even with the use of masks, most of the respondents did not feel better about teeth and smile esthetics, suggesting that esthetics is important for themselves. Most participants reported missing to look at people’s smiles since they consider it important. Facial expression plays an important role in social interaction, with the use of mask, many subtle facial expressions are lost, and the smiles remain unseen, with the loss of facial communication. Blais *et al.* (25) found that the mouth area is the most important cue for both static and dynamic facial expressions. Emotional facial expressions, including ganger, fear, surprise, sadness, happiness, and disgust, may not be detected with mask use, decreasing social communication and impacting the importance of visualizing the mouth area. Women missed significantly more looking at other people’s smiles. This result probably is correlated with the finding that women are positively associated with modern health concerns (26).

Female respondents were the majority in the present study. It was not surprising since women are more willing to participate in surveys and researches (10,22,27,28).

In this open survey, it was not possible to calculate the response rate. Open surveys generate interesting data (even if not necessarily generalizable), in particular when a qualitative analysis is the aim and the objective is to study current trends in a specific issue (29), like in the present study. Regarding the representativeness of the sample, the internet community is becoming more representative of society as a whole (30). This way, the number of participants and the representativeness of the sample were quite sufficient and suiTable, allowing reliable conclusions to the objectives of this study.

A possible limitation of the present study was the representativeness of the sample of convenience that does not allow population inferences or generalizations. However, online surveys allow easier access and preserve participants’ anonymity, reducing the tendency of socially desirable responses. Even so, the methodology expanded the sample to all Brazilian regions and covered all age ranges. As far as we know, these data were not yet reported in the literature, and it is strongly important, due to the particularities of the current situation.

The results of this study are a substantial contribution to a field with scarce research. This research has relevant implications for oral health, since the decrease of toothbrushing frequency may cause deterioration of the population’s oral conditions. Moreover, dentists may know the current patient’s motivations and intention to seek dental treatment. Cultural and timely differences may occur and suggest the need for current studies in other countries and populations.
